# The COVID-19 pandemic: research and health development in the World Health Organisation Africa region

**DOI:** 10.11604/pamj.supp.2020.35.2.23628

**Published:** 2020-05-27

**Authors:** Matshidiso Moeti, Joseph Cabore, Francis Kasolo, Zabulon Yoti, Felicitas Zawaira, Moredreck Chibi, Soatiana Rajatonirina, Humphrey Karamagi, Helen Rees, Richard Mihigo, Michel Yao, Benido Impouma, Joseph Chukwudi Okeibunor, Ambrose Otau Talisuna

**Affiliations:** 1 WHO Regional Office for Africa, Brazzaville, Congo; 2 Witwatersrand Reproductive Health and HIV Institute, South Africa

**Keywords:** COVID-19, research, health development, WHO, African region

## Abstract

Concerns have been expressed about the view point of WHO AFRO concerning research for health in the African Region. WHO AFRO considers research a critical component in the improvement of health in the Africa region. Ensuring the effectiveness of our strategies, policies and programmes requires evidence. In the context of the ongoing COVID-19 outbreak, WHO research interests cover key areas of the response. The WHO AFRO consider research as critical in our efforts at protecting people against health emergencies and pandemics like the COVID-19 and ensuring universal access to proven interventions. In view of this, the WHO has taken steps to strengthen capacity for research in the region. The results of these efforts may take time to manifest but will surely do as we persist in our drive, with support from our partners.

## Perspective

Our attention has been drawn to some recent commentaries on the need for research tailored to the realities of the African people in the context of COVID-19. In her contribution to the Guardian, Dr Monique Wasunna, director of DNDI´s Africa regional office, warned that if research is not conducted in Africa, potential life-saving innovations will be delayed [1]. Similarly, writing in Quartz Africa, Gale Ure, a researcher from Witwatersrand, noted the importance of legitimate medical research activities to ensure pandemics like the COVID-19 tragedy can be managed [2]. We agree with these viewpoints. WHO AFRO considers research a critical component in our actions to improve health in the Africa region. To ensure our strategies, policies and programmes are effective, they must be based on, and informed by, evidence. At WHO research for health spans 5 generic areas of activity as part of our response to the ongoing COVID-19 outbreak. These include measuring the magnitude and distribution of the health problem; understanding the diverse causes or the determinants of the problem, whether they are due to biological, behavioural, social or environmental factors; developing solutions or interventions that will help to prevent or mitigate the problem; implementing or delivering solutions through policies and programmes; and evaluating the impact of these solutions on the level and distribution of the problem.

Our top priority is to support Member States to achieve universal health coverage as part of the collective action to achieve the Sustainable Development Goals based on the Primary Health Care approach. Our organization-wide strategic plan for achieving this, is the Thirteenth General Programme of Work (GPW13) 2019-2023. The main thrust of this guiding document is to promote health, keep the world safe, serve the vulnerable, by delivering on three triple billion targets: 1) Achieving universal health coverage, with 1 billion more people benefiting from universal health coverage; 2) Addressing health emergencies, with 1 billion more people better protected from health emergencies; 3) Promoting healthier populations, with 1 billion more people enjoying better health and well-being. Research and innovation occupy a central position in the GPW13, to focus global public goods on impact and ensure that WHO is a knowledge-based organization facilitating normative guidance, sharing data and scaling up innovations.

The ongoing COVID-19 pandemic has further unraveled the urgent need for therapeutics, vaccines, diagnostics and other public health interventions. This leaves the medical and public health community with only non-pharmaceutical interventions (NPIs) to rely on for reducing the burden of COVID-19. Improving our response to the COVID-19 Pandemic in the WHO Africa region requires innovation, new information and research and development (R and D) is urgently needed. The SARs-CoV-2 virus is a new pathogen that spreads quickly, and is causing enormous health, economic and societal impacts around the world. Response to this pandemic requires a multi-sectoral approach, guided by critical knowledge on the diverse dimensions of the disease. Dr Tedros A. Ghebreyesus, WHO´s Director General has urged that, *“we need our collective knowledge, insight and experiences to answer the questions we don´t have answers to, and to identify the questions we may not even realize we need to ask”*. In recognition of these facts, we have taken steps to enhance the conduct of research in the region. A recent national health research systems assessment in the region revealed gaps in many countries in terms of research norms, infrastructure, and funding. These are gaps that need to be filled urgently as we strive to conduct research in the region [3,4].

We considered this step critical for three main reasons: (1) governments and health care leaders should know if there are unique aspects about how these interventions may work in their populations and health care systems; (2) there is a moral imperative for Africa to be part of the global initiative to find solutions to any pandemic; and (3) the recognition that it will be more difficult to advocate for access to effective products if countries/regions have not played their part in trying to determine which products are effective. Response to the COVID-19 pandemic requires action beyond the health sector and simply adopting strategies only based on well-known pathogens, risks failing to exploit all possible measures to slow transmission of the virus. This calls for research to answer various questions and develop innovative approaches in response to the pandemic.

Major priorities for research include:

**Hosting a hackathon in the African region:** leveraging the database of innovators from continent through several innovation hubs and accelerators across the continent, WHO AFRO shall call for innovations that are suitable for the African region context to help respond to the current COVID-19 response.

**Online innovation market platform for COVID-19:** develop a web-based platform for collating innovation submissions for COVID-19. The platform will be accessible to Member States to identify appropriate innovative solutions that they will adopt and scale in their respective countries. A review committee will be constituted to periodically review the science and maturity of the innovations, and their potential impact to develop a pipeline fit for adoption and scale in the Region.

**Case studies for innovations developed in other countries globally:** implement a systematic approach to identify the innovations that were developed in other regions, for instance in China and Europe. Perform implementation research and gain knowledge on whether those solutions can be adapted to the current African context.

**Early investigations of COVID-19 cases in countries:** engage with countries to introduce the five WHO standard protocols for early investigation of COVID-19 cases in the Region. Give orientation to countries that indicate interest on any or all the five protocols. Provide both technical and financial support on the implementation of the selected protocols.

**Research on priority questions around COVID-19:** facilitate the setting up of research teams for the response. Share the research priorities with country response teams and partners, facilitate coordinated research actions, Facilitate the use of generic protocols and finally, mobilize resources to support implementation of research protocols in countries.

In collaboration with partners, such as the African Academy of Science (AAS); the European and Developing Countries Clinical Trial Partnership (EDCTP); the Coalition for Epidemic Preparedness and Innovations (CEPI); among others, we have taken steps to develop clinical trial regulatory oversight capacity over the years. For instance, the African Vaccine Regulatory Forum (AVAREF) was established as a mechanism to assure the quality of reviews through the technical support of WHO, African experts and other experts, as needed. The WHO in African region has also produced guidance documents for use by countries in building their research infrastructure.

There are some major research outcomes that have contributed to change in the way we do things in the region that WHO has been involved in. For example, WHO was at the center of the development of the AFR meningitis A vaccine which has exclusively been used in Africa and has stopped outbreaks of meningitis due to this serotype [5,6]. WHO in the African Region, through the AVAREF secretariat, housed in WHO Regional Office for Africa, played and continues to play very pivotal roles in in Malaria vaccine [7] and recently the Ebola vaccine. WHO, in collaboration with partners, including PATH, a non-profit organization, and GSK, the vaccine manufacturer, supported the ministries of health in each of the pilot countries to leading vaccine introduction. Each partner has clearly defined roles: WHO was responsible for coordination of the MVIP and provided scientific and technical leadership for the programme; WHO also supports the ministries of health as they introduce the vaccine [8].

With respect to Ebola vaccine, WHO played major roles, from supporting the research, to conducting the trial in Guinea in 2015. A randomized trial for the vaccine began during the West Africa Ebola outbreak in 2015. With support from WHO, the government of Guinea and WHO took the unusual step to lead the trial, when no other organization was positioned to run a trial in Guinea during the complex emergency. During the current Ebola outbreak in the Democratic Republic of the Congo, more than 236,000 people were vaccinated with rVSV ZEBOV GP donated by Merck to WHO, including more than 60,000 health and frontline workers in the Democratic Republic of the Congo and in Uganda, South Sudan, Rwanda and Burundi. Furthermore, for the past five years, WHO has consistently convened experts to review the evidence on various Ebola vaccine candidates, informed policy recommendations, and mobilized a multilateral coalition to accelerate clinical evaluations. The EMA review was unique in that WHO and African regulators actively participated through an innovative cooperative arrangement put in place by WHO, which will help accelerate registration for the countries, most at risk [9].

With respect to diagnostics, WHO in the African Region has major strides. For example, WHO supported evidence informed tools and policy development and at least 20 new technologies were developed and evaluated in the last decade [10]. Further, in October 2019, WHO reconstituted the African Advisory Committee on Health Research and Development to play a vital role in advancing health research in our Region. In reconstituting the advisory committee, we were mindful of the need to strengthen our leadership role in shaping research priorities and agenda in the Region, and ensuring that research capacities can be enhanced, and persistent gaps addressed. We were also mindful of the need to support the generation and use of research to guide countries in moving forward towards universal health coverage within the framework of the Sustainable Development Goals.

## Conclusion

We would like to reiterate that research is critical for our work as an organization. It is a core function of the WHO in shaping the research agenda and stimulating the generation, translation and dissemination of valuable knowledge. We consider research critical for progressing on the UHC and SDG agenda, and more importantly protecting the people against health emergencies and pandemics like the COVID-19. Consequently, the WHO has taken steps to strengthen capacity for research in the region. The results of these efforts may take time to manifest but will surely do as we persist in our drive, with support from our partners.

## Competing interests

The authors declare no competing interests.
